# Transcriptomic Approach Reveals Contrasting Patterns of Differential Gene Expression during Tannin Biodegredation by *Aspergillus tubingensis* in Liquid and Solid Cultures

**DOI:** 10.3390/ijms251910547

**Published:** 2024-09-30

**Authors:** Xiaona Zeng, Jiabei Song, Shengqiu Tang, Xiaoying Dong, Sheng Chen, Jie Kong, Liyi Chen, Yajuan Li, Guanming Shao, Yung-Hou Wong, Qingmei Xie

**Affiliations:** 1 State Key Laboratory of Swine and Poultry Breeding Industry & Heyuan Branch, Guangdong Provincial Laboratory of Lingnan Modern Agricultural Science and Technology, College of Animal Science, South China Agricultural University, Guangzhou 510642, China; xiaona.zeng@scau.edu.cn (X.Z.);; 2 Henry Fok School of Biology and Agriculture, Shaoguan University, Shaoguan 512005, China; 3 Guangdong Provincial Key Lab of Agro-Animal Genomics and Molecular Breeding, College of Animal Science, South China Agricultural University, Guangzhou 510642, China; 4 Guangdong Engineering Research Center for Vector Vaccine of Animal Virus, Guangzhou 510642, China; 5 Division of Life Sciences, Biotechnology Research Institute, Hong Kong University of Science and Technology, Hong Kong, China

**Keywords:** *Aspergillus tubingensis*, solid culture, liquid culture, transcriptomic, tannin biodegredation

## Abstract

Tannins, one of the most common anti-nutritional factors in feed, can be effectively degraded by various enzymes secreted by *Aspergillus tubingensis* (*A. tubingensis*). The cultivation method of fungi significantly impacts gene expression, which influences the production of enzymes and metabolites. In this study, we analyzed the tannin biodegredation efficiency and the transcriptomic responses of *A. tubingensis* in liquid and solid cultures with tannin added. The observed morphology of *A. tubingensis* resembled typical fungal hyphae of mycelium submerged and grown in liquid cultures, while mainly spore clusters were observed in solid cultures. Furthermore, the tannin biodegredation efficiency and protein secretion of *A. tubingensis* in liquid cultures were significantly higher than in solid cultures. Additionally, 54.6% of the 11,248 differentially expressed genes were upregulated in liquid cultures, including AtWU_03490 (encoding ABC multidrug transporter), AtWU_03807 (ribonuclease III), AtWU_10270 (peptidyl-tRNA hydrolase), and AtWU_00075 (arabinogalactan endo-1,4-beta-galactosidase). Functional and gene ontology enrichment analyses indicated upregulation in processes including oxidation reduction, drug metabolism, and monocarboxylic acid metabolism. Overall, this study provides insight into the transcriptomic response to tannin biodegradation by *A. tubingensis* in different cultures and reveals that liquid cultures induce greater transcriptomic variability compared to solid cultures.

## 1. Introduction

Tannins are polyphenols synthesized during a plant’s secondary metabolism that can directly or indirectly affect intake and digestion and have long been known to be an anti-nutritional agent in monogastric animals, poultry, and aquatic animals [1,2,3,4]. Thermal treatments and non-thermal treatments (such as fermentation and enzymatic treatment) are identified as methods to reduce the levels of anti-nutrients [5,6,7]. Over the years, microbial tannin-active enzyme-catalyzed tannin degradation has been considered a promising approach for tannin biodegredation [8,9,10,11]. Filamentous fungi can secrete enzymes for tannin biodegredation [12,13,14]. *Aspergillus tubingensis* (*A. tubingensis*), a common food-grade filamentous fungus, could be used as a source of biodegradation enzymes [15,16,17]. However, the mechanisms of tannin biodegredation by *A. tubingensis* are not yet fully understood.

The cultivation method of fungi significantly impacts gene expression, influencing the production of enzymes and metabolites [18]. Solid cultures have been employed in the food industry to optimize the production of secondary metabolites, while liquid cultures are favored at the laboratory scale for studying fungal responses to different stimuli in nature [19,20]. Secondary metabolites produced by members of the *Aspergillus* genus are primarily generated in solid culture media. However, extracting fungal biomass from solid culture media is challenging [21]. In solid cultures, colonies encounter changing substrates as mycelial growth progresses. Conversely, in liquid cultures, mycelia are exposed to a more homogeneous substrate utilized over time, resulting in various mycelial forms ranging from dispersed mycelia to dense clumps as time progresses [22]. However, whether and to what extent this culture method influences the production of enzymes and metabolites in fungi has not been studied in depth.

Transcriptomics, in particular, can reveal the biological effects of various environmental factors on gene expression [23,24,25]. It has been reported that the transcriptomic response of *Aspergillus niger* to solid-state fermentation is influenced by the culture method used, limiting the value of liquid cultures in understanding fungal behavior in natural environments [26]. However, a comprehensive understanding of the transcriptional regulatory mechanisms governing tannin biodegredation by *A. tubingensis* is still lacking and warrants further investigation.

In this study, we compared the transcriptomic responses of *A. tubingensis* to tannin biodegredation under solid and liquid culture conditions to identify genes directly or indirectly related to tannin biodegredation, and to improve knowledge of tannin biodegredation of *A. tubingensis*.

## 2. Results

### 2.1. The Morphology of A. tubingensis in Different Cultures

**Figure 1:**
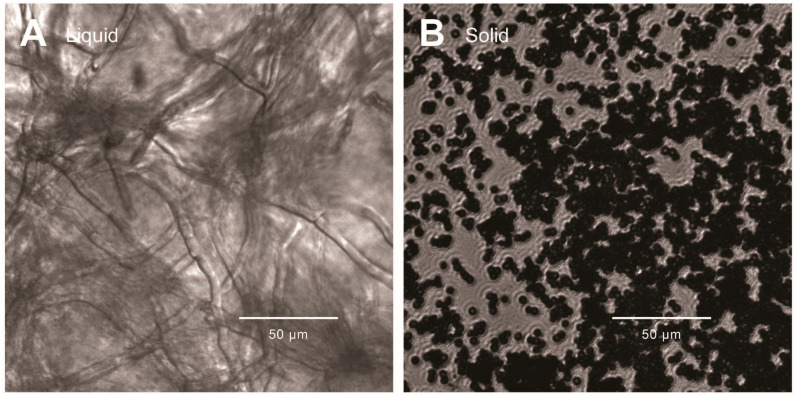
Growth morphology of *A. tubingensis* in different culture conditions. (**A**) The morphology of *A. tubingensis* grown in liquid cultures with tannin added. (**B**) The morphology of *A. tubingensis* grown in solid cultures with tannin added. The scale bar indicates 50 µm.

The growth morphology of *A. tubingensis* showed significant differences under different culture conditions. In liquid cultures, the morphology of *A. tubingensis* resembled typical fungal hyphae of submerged growing mycelium (Figure 1A), whereas in solid cultures, it primarily exhibited spore clusters (Figure 1B).

### 2.2. The Efficiency of Tannin Degradation by A. tubingensis in Different Cultures

**Figure 2:**
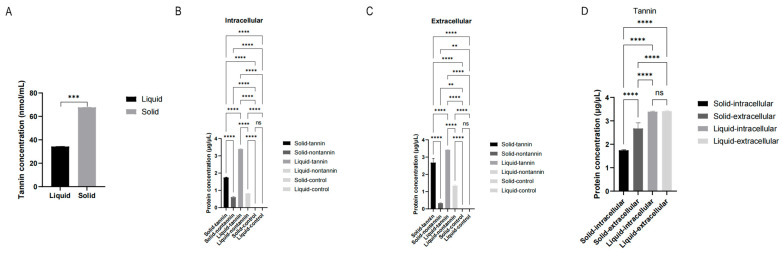
The efficiency of tannin biodegredation and protein secretion by *A. tubingensis* in different culture conditions. (**A**) Final tannin concentration of *A. tubingensis* in different culture conditions. (**B**) The concentration of intracellular proteins secreted by *A. tubingensis* in liquid or solid cultures with or without tannin. (**C**) The concentration of extracellular proteins secreted by *A. tubingensis* in liquid or solid cultures with or without tannin. (**D**) The concentration of intracellular and extracellular proteins secreted by *A. tubingensis* in liquid or solid cultures with tannin. Liquid, *A. tubingensis* in liquid cultures. Solid, *A. tubingensis* in solid cultures. Liquid-control, non-*A. tubingensis* in liquid cultures. Solid-control, non-*A. tubingensis* in solid cultures. ns, *p* > 0.05. **, *p* < 0.01. ***, *p* < 0.001, ****, *p* < 0.0001.

To assess the efficiency of tannin degradation by *A. tubingensis* under different cultivation conditions, tannin concentrations were measured after 96 h of culturing. The initial tannin concentration was 10 g/L in both liquid and solid cultures. After 96 h culturing, the final tannin concentration in the liquid cultures was significantly lower than that of solid cultures (Figure 2A), indicating a higher efficiency of tannin degradation by *A. tubingensis* in liquid cultures compared to solid cultures.

### 2.3. The Protein Concentration Secreted by A. tubingensis in Different Cultures

The concentration of intracellular and extracellular proteins secreted by *A. tubingensis* under different cultivation conditions was determined after 96 h of culturing. The intracellular protein concentration (Figure 2B) and extracellular protein concentration (Figure 2C) in both liquid and solid cultures containing tannin were significantly higher than those in cultures without tannin, indicating that tannin induces more secretion of both intracellular and extracellular proteins by *A. tubingensis*. Furthermore, the intracellular protein concentration (Figure 2B) and extracellular protein concentration (Figure 2C) in liquid cultures were significantly higher than those in solid cultures, indicating that liquid cultures enhance protein secretion. Additionally, tannin induced significantly more secretion of extracellular proteins compared to intracellular proteins in solid cultures, while in liquid cultures, there was no significant difference between the concentrations of extracellular and intracellular proteins induced by tannin (Figure 2D).

### 2.4. The Transcriptomic Responses to Tannin Biodegredation of A. tubingensis

**Figure 3:**
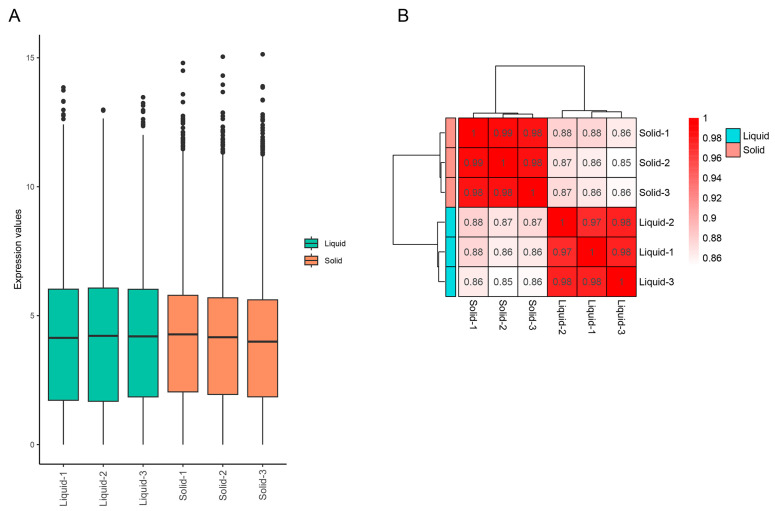
Distribution of gene expression of *A. tubingensis* in different culture conditions. (**A**) Boxplot for all genes of *A. tubingensis* in liquid and solid cultures. Different colors indicate different culture conditions that were used in *A. tubingensis*. The black dots indicate outlier genes with extremely low or high expression values. (**B**) Correlation heatmap analysis of *A. tubingensis* in liquid and solid cultures. Color intensity is related to the numbers within them. Liquid, *A. tubingensis* in liquid cultures. Solid, *A. tubingensis* in solid cultures.

**Figure 4:**
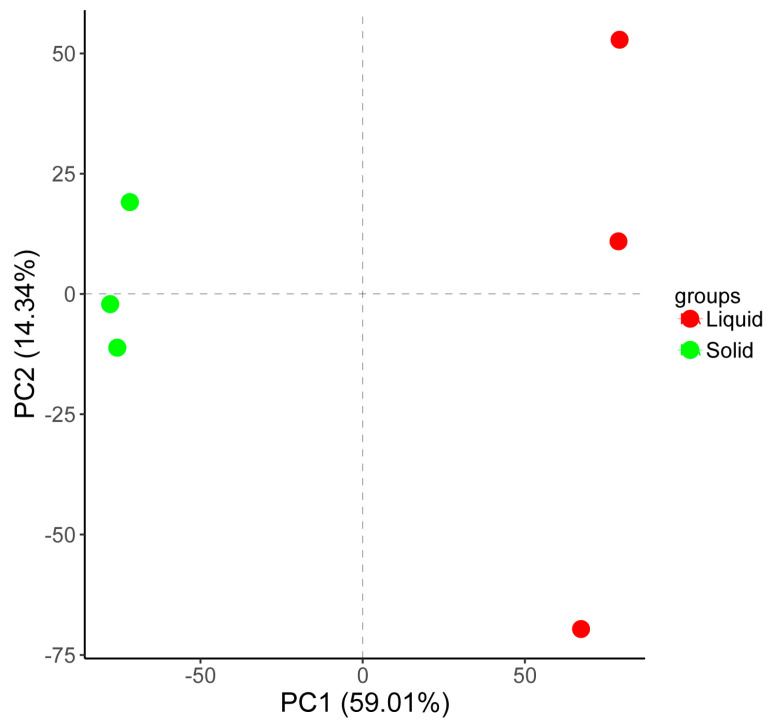
Principal component analysis (PCA) of *A. tubingensis* in different culture conditions. PCA identified two clusters in the data separated along the second and third principal components. The percentages on each axis represent the percentages of variation explained by the principal components. PC1 and PC2 define 59.01% and 14.34% of the variance, respectively. The distance between the points reflects the variance in gene expression between them. Liquid, *A. tubingensis* in liquid cultures. Solid, *A. tubingensis* in solid cultures.

**Figure 5:**
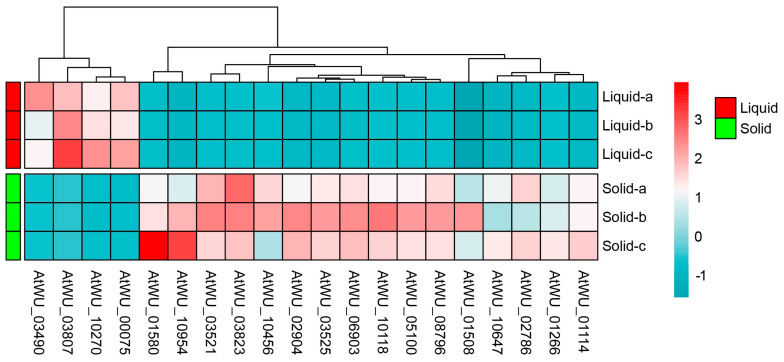
Heatmap of DEGs for comparing *A. tubingensis* grown in liquid and solid cultures. Genes on the heatmap are organized by hierarchical clustering based on the similarity in expression patterns. Liquid, *A. tubingensis* in liquid cultures. Solid, *A. tubingensis* in solid cultures.

**Figure 6:**
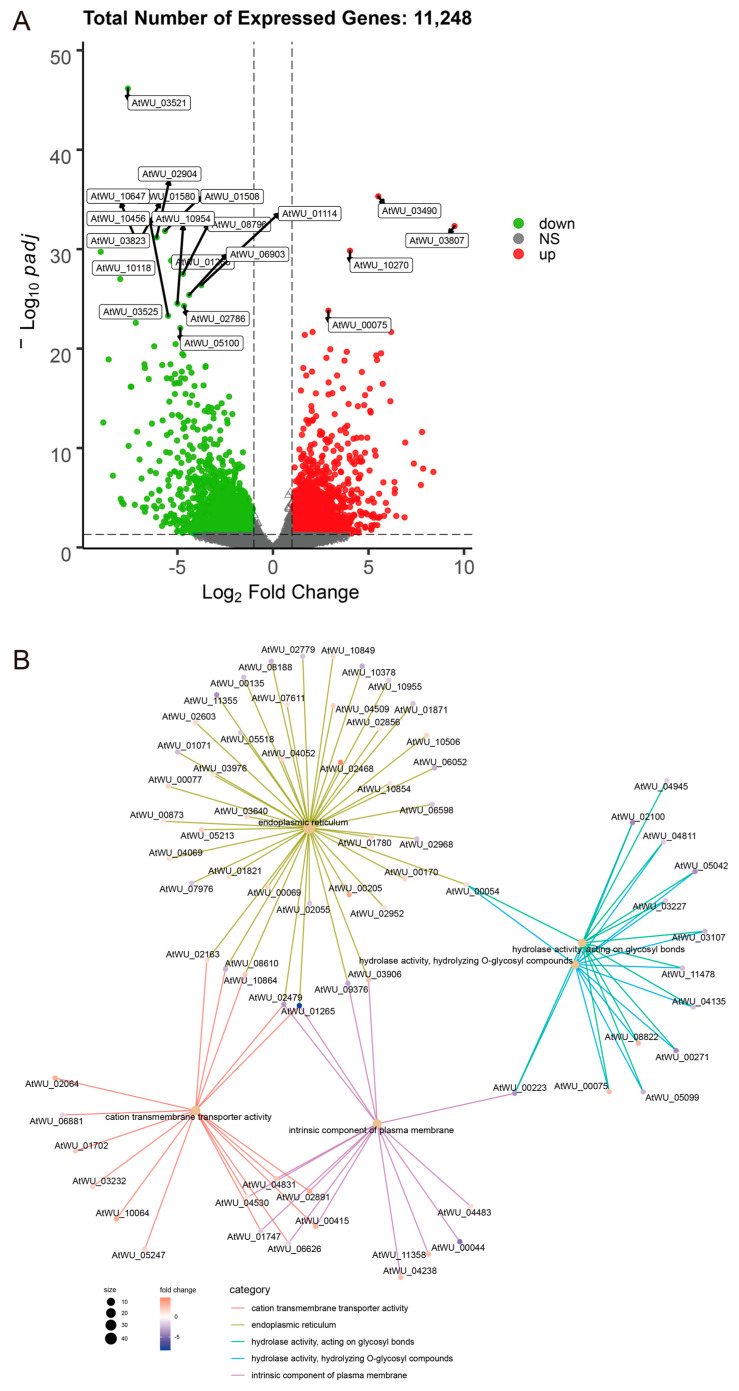
DEGs and functional enrichment. (**A**) Volcano plot showing the top 20 DEGs between liquid and solid cultures. The volcano plots distribution of log fold change (x-axis) and the negative log (base 10) of the *p*-values (y-axis). The data points above the significance threshold (q < 0.05, foldchange > 2) are marked in red (upregulated genes) and green (downregulated genes). (**B**) The gene cnetplot network of *A. tubingensis* grown in liquid and solid cultures.

A transcriptomic assay was performed to characterize the responses of *A. tubingensis* to tannin biodegredation under different cultivation conditions. Boxplots revealed significant differences in the distribution of gene expression levels between liquid and solid cultures (Figure 3A). The intensity of expression and patterns of these genes varied significantly between the two culture conditions (Figure 3B). Notably, principal component analysis (PCA) score plots showed distinct clusters for liquid and solid cultures, indicating different gene expression patterns under these conditions (Figure 4). A heatmap clustering analysis further revealed significant differences in the expression of 20 genes between the two culture conditions (Figure 5), highlighting the distinct impacts of liquid and solid cultures on overall gene expression. Additionally, a volcano analysis identified 11,248 differentially expressed genes (DEGs) in *A. tubingensis* between the two cultures, with four genes (AtWU_03490, AtWU_03807, AtWU_10270, and AtWU_00075) significantly upregulated in liquid cultures, and one gene (AtWU_03521) significantly downregulated (Figure 6A). The upregulated gene AtWU_00075 in liquid cultures is related to hydrolase activity (Figure 6B). The upregulated gene AtWU_03490 is related to ABC multidrug transport, AtWU_03807 is related to Ribonuclease III enzymes that are responsible for processing RNA precursors into functional RNAs, and AtWU_10270 is related to the PeptidyI-tRNA hydrolase that releases tRNA from peptidyI-tRNA. The downregulated gene AtWU_03521 is related to the mammalian suppressor of Section 4, a regulator of stress responses and apoptosis.

A Gene Set Enrichment Analysis (GSEA) and an Over-Representation Analysis (ORA) of the DEGs were conducted to determine the biological processes in *A. tubingensis* within liquid and solid cultures. For GSEA, the top 10 enriched gene ontology (GO) terms associated with tannin degradation in liquid culture included processes related to protein organization, nucleic acid regulation, RNA biosynthesis, and metabolic regulation (Table 1). For ORA, enriched processes in liquid cultures included DNA metabolism, RNA biosynthesis, and aromatic compound biosynthesis (Table 1). Additionally, a Kyoto Encyclopedia of Genes and Genomes (KEGG) analysis was performed, which showed enrichment in pathways related to the biosynthesis of secondary metabolites in eukaryotes, microbial metabolism in diverse environments, and biosynthesis of cofactors. The ORA KEGG analysis revealed associations with pathways such as the biosynthesis of cofactors, amino acid biosynthesis, the yeast cell cycle, carbon metabolism, protein processing, peroxisomes (spliceosomes), and starch and sucrose metabolism (Table 2).

### 2.5. Validation of RNA-Seq Results by RT-qPCR

**Figure 7:**
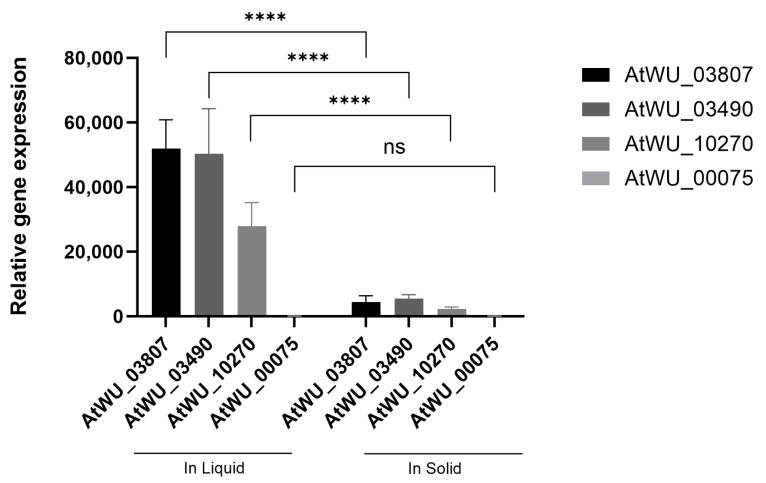
Gene expression patterns of the selected four DEGs. Gene expression profile of the top four genes in *A. tubingensis* grown in liquid cultures compared with those in solid cultures. The y-axis shows the mRNA levels of gene expression. ns, *p* > 0.05. ****, *p* < 0.0001.

RT-qPCR was performed to validate the most significantly upregulated genes in both solid and liquid cultures. The β-tubulin was used as the reference gene. Upregulated DEGs (AtWU_03490, AtWU_03807, AtWU_10270, and AtWU_00075) from RNA-Seq were selected for RT-qPCR, and the results showed that AtWU_03490, AtWU_03807, and AtWU_10270 had significantly higher expression in liquid cultures compared to solid cultures (Figure 7).

### 2.6. Confirmation of Transcription Factor by Dual-Luciferase Reporter Assay

**Figure 8:**
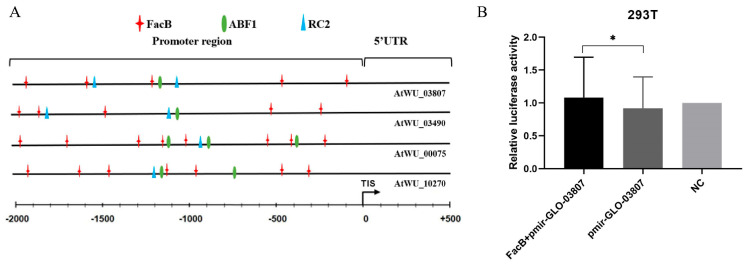
Transcriptional activation by FacB. (**A**) Locations of transcription factor binding sites on promoter regions of four differentially expressed genes were predicted by the MatInspector program. TIS, translational initiation site; 5′-UTR, 5′-untranslated region. (**B**) The dual-luciferase reporter assay confirmed the impact of FacB on promoter function. NC, negative group without transfected. FacB + pmir-GL0-03807, cells transfected with plasmid FacB and pmir-GL0-03807. pmir-GL0-03807, cells transfected with plasmid pmir-GL0-03807. *, *p* < 0.05.

The JASPAR database of transcription factor binding profiles (www.jasper.genereg.net) predicted that the promoter sites of the four most upregulated genes (AtWU_03807, AtWU_03490, AtWU_00075, and AtWU_10270) would contain binding sites for the transcription factor FacB (Figure 8A). To further confirm the effect of FacB on the promoter function of gene AtWU_03807, a dual-luciferase reporter assay was conducted. The results revealed that pRK5-cMYC-FacB-transfected cells exhibited greater promoter function compared to those transfected with the negative plasmid (Figure 8B).

### 2.7. Formatting of Mathematical Components

Not applicable.

## 3. Discussion

As an anti-nutritional agent, tannins can be biodegradated by tannin-active enzymes secreted from *Aspergillus* [2,16]. However, the regulatory mechanisms of tannin biodegredation by *Aspergillus* remain unclear. Whether and to what extent the cultivation method influences the production of enzymes and metabolites in *Aspergillus* have not been studied in detail. In this study, transcriptomic responses related to tannin biodegredation in *Aspergillus* under liquid and solid culture conditions were analyzed to assess the potential mechanisms of tannin biodegredation by *Aspergillus*.

Utilizing tannins as a complex substrate requires a variety of biodegrading enzymes. Filamentous fungi produce a range of hydrolytic and oxidative enzymes that enable them to grow on diverse and complex plant biomass [27,28]. In the present study, the distinct growth morphology and protein secretion under different cultivation conditions suggest that the tannin biodegredation pathways of *A. tubingensis* may differ. When adding tannin into the culture media, the intracellular and extracellular protein concentrations of *A. tubingensis* in liquid cultures were significantly higher than those in solid cultures. This result was similar to previous research on the filamentous fungus *Phanerochaete chrysosporium*, which showed a higher quantity of enzymes involved in carbohydrate-binding modules under liquid culture conditions compared to solid culture conditions [19,22]. Previous studies have also indicated that both gene expression and enzyme production can vary in different parts of the mycelium [26,29]. Aspergilli may produce various small, secreted proteins including hydrophobins, hydrophobic surface-binding proteins, and effector proteins to decompose solid polymers [30]. Hydrophobins contribute to the formation of aerial hyphae and conidia [31]. The expression profiles of multiple hydrophobin genes depend on the growth stage of filamentous fungi and culture conditions [32,33].

Understanding the potential metabolic mechanisms responsible for inducing and maintaining desired fungal structures in a medium is crucial for inducing specific fungal structures and overcoming bottlenecks in industrial fungal production [28]. To gain a better understanding of the transcriptomic response of *A. tubingensis* to tannin biodegredation, the DEGs related to tannin biodegredation in cells grown under solid and liquid culture conditions were analyzed. Gene expression variability was found to be lower in solid cultures compared to liquid cultures. The four upregulated DEGs in liquid cultures were related to gene encoding, ABC multidrug transporters, ribonuclease III, peptide tRNA hydrolase, and arabinoxylan-1,4-beta-xylosidase. ABC multidrug transporters typically display high basal ATPase activity, which is often moderately stimulated by drugs in bacterial transporters [34,35]. ATP, a high-energy phosphate compound, ensures the energy supply for various cellular activities [36]. Fungi require energy for transcription, secretion of small acidic compounds, and biodegradation [24]. Ribonuclease III enzymes are responsible for processing RNA precursors into functional RNAs that participate in protein synthesis, RNA interference, and a range of other cellular activities [37]. Peptidyl-tRNA hydrolase plays a critical role in protein biosynthesis [38]. Arabinoxylan-1,4-beta-xylosidase is an enzyme that degrades cellulose [39]. In addition, the promoter sites of these four DEGs were predicted to bind various transcription factors, including FacB, RC2, and ABF1. The activation of FacB implies that *A.tubingensis* grown on tannin as the main carbon source also needs to utilize host carbon sources, such as acetate, under the regulatory control of transcription factor FacB [40,41,42]. Available extracellular carbon sources, such as glucose and acetate, can significantly affect *A. fumigatus*’ secondary metabolite secretion and cell wall composition [42]. The differences in DEGs of *A. tubingensis* under solid and liquid culture conditions may be attributed to various environmental factors to which the fungus is exposed, such as aeration, oxygen diffusion rate, osmotic pressure, viscosity, spore inoculum density, growth type, or substrate availability, which can vary significantly between solid and liquid culture conditions [43,44].

A GO enrichment analysis revealed enrichments in categories such as DNA metabolic processes, RNA processes, and protein biosynthetic processes in liquid cultures. Specifically, DNA metabolic processes, including DNA damage, stimulus, and repair processing, were enriched in liquid cultures, indicating oxidative stress induced by tannins in *A. tubingensis* grown under liquid cultures. This finding aligns with the recognized mutagenic properties of aromatic and other poly-aromatic compounds [45,46]. Furthermore, the top 10 KEGG results were analyzed and found to be enriched in liquid cultures, including biosynthesis of secondary metabolites, protein processing in the endoplasmic reticulum, carbon metabolism, biosynthesis of amino acids, and starch and sucrose metabolism. This could be attributed to the faster depletion of other preferred carbon sources in the liquid medium compared to solid medium due to the higher availability of the (partially) dissolved substrates [47,48]. These findings hold significant implications for the industry, as liquid cultures are favored over solid cultures in enzyme production due to better control of sterile conditions and process management.

## 4. Materials and Methods

### 4.1. Strain and Culture Conditions

The fungal strain used in this study was *Aspergillus tubingensis* TPDA-1, obtained from South China Agricultural University. Initially, the strain was cultivated in a basic culture medium (BCM) with a pH of 5.5, consisting of potato extract (4 g/L) and glucose (20 g/L). After 96 h of cultivation at 30 °C, the resulting spores from the strain in the BCM supplemented with 18% ager were harvested and measured with a counting chamber (Countess^TM^ 3 Automated Cell Counter, Thermos Fisher Scientific, Inc., Waltham, MA, USA).

The liquid culture medium consisted of 50 mL BCM combined with 5 g tannin powder (Aladdin, Shanghai, China). For the liquid culture medium, 5 g tannin powder was added into 5 mL ddH_2_O to prepare for tannin stock solution, and could be dissolved after autoclaving. The solid culture medium consisted of 50 mL BCM with 5 mL tannin stock solution. For both the liquid and solid culture media, the pH was confirmed to be 5.5 after the addition of tannin.

For liquid cultures, 1 mL of spores (1 × 10^7^/mL) was inoculated into 50 mL of liquid culture medium and cultured at 30 °C and at 250 rpm on a shaker for 96 h. For solid cultures, 1 mL of spores (1 × 10^7^/mL) was cultured in 50 mL of solid culture medium and cultured at 30 °C for 96 h. For the control group, 1 mL of sterilized saline was added into 50 mL of BCM medium supplemented with 10% tannin and cultured at 30 °C for 96 h. Three replicates of each group were performed. After cultivation, mycelia were harvested and stored at −80 °C for subsequent experiments.

### 4.2. Mycelia Harvest and Electron Microscopy Observation

*A. tubingensis* in liquid cultures was centrifuged at 12,000 rpm for 10 min at room temperature to precipitate mycelia for subsequent analysis. *A. tubingensis* in solid cultures were resuspended in PBS and then centrifuged at 12,000 rpm for 10 min at room temperature to precipitate mycelia for subsequent analysis. The harvested mycelia of *A. tubingensis* were resuspended in PBS, spotted onto microscope slides, and visualized under a Talos F200 transmission electron microscope (Talos F200S; Thermos Fisher Scientific, Inc., Waltham, MA, USA).

### 4.3. Tannin and Protein Quantification Assays

An amount of 1 mL of *A. tubingensis* spores (1 × 10^7^) was cultured in solid and liquid cultures at 30 °C for 96 h, respectively. Subsequently, *A. tubingensis* in liquid cultures was centrifuged at 12,000 rpm for 10 min at room temperature. *A. tubingensis* in solid cultures was resuspended in PBS and then centrifuged at 12,000 rpm for 10 min at room temperature. Next, the supernatant was collected for extracellular protein concentration measurement. The precipitates were collected for intracellular protein concentration measurement. Intracellular and extracellular protein concentrations of *A. tubingensis* were analyzed using the Pierce^TM^ Rapid Gold BCA protein assay kit (Thermos Fisher, USA) according to the manufacturer’s protocol. The tannin content was determined using a tannin content assay kit (Boxbio Science & Technology, Beijing, China) with measurement taking place at a wavelength of 320 nm using ultraviolet spectrometry.

### 4.4. RNA Extraction and Transcriptome Sequencing

The total RNA was extracted from the mycelia of *A. tubingensis* (40–50 mg) using the RNeasy Plant Mini kit (Qiagen, Beijing, China) following the provided protocol. Briefly, frozen mycelia of *A. tubingensis* were placed in 2 mL microcentrifuge tubes precooled in liquid nitrogen, and ground to a powder at 5 °C for 10 min using a Retsch Mixer. The RNA concentration was determined by a UV absorbance of 260 nm, and the RNA purity was assessed using a Nanophotometer^®^ spectrophotometer (IMPLEN, Westlake Village, CA, USA). The RNA integrity was evaluated using the RNA Nano 6000 Assay Kit on the Bioanalyzer 2100 system (Agilent Technologies, Santa Clara, CA, USA). For library preparation, 1 µg of RNA per fungal sample was used as input material. Transcriptome sequencing was performed using the Illumina^®^ HiSeq2000 system as previously described [49]. The workflow included rRNA removal, RNA fragmentation, double-stranded cDNA synthesis, adenylation of the 3′ ends, adapter ligation, PCR amplification, library quality assessment, and sequencing on the NextSeq550 next-generation sequencing platform. Briefly, the first strand of cDNA was synthesized using fragmented mRNA as a template and random oligonucleotides as primers, followed by degradation of the RNA strand by RNaseH (ribonuclease H) (Thermos Fisher, USA). The second strand of cDNA was synthesized from dNTPs (four kinds of deoxyribonucleotides) in the DNA polymerase I system (Thermos Fisher, USA). The purified double-stranded cDNA was end-repaired, and then an adenylate tail was added and ligated with the sequencing adapter. An amount of 250–300 bp cDNA was screened and amplified by PCR. In brief, 20 µL reaction mixtures were prepared and performed at 95 °C for 10 min; 94 °C for 30 s, 55 °C for 1 min with 32 cycles; and 98 °C for 10 min. PCR products were purified by AMPure XP magnetic beads (Thermos Fisher, Inc., Waltham, MA, USA). Clean reads were obtained by removing adapter sequences from the raw RNA-seq reads. The quality of the clean reads was analyzed using FastQC software version 0.11.9, and the reads were stored in the FASTQ file format. Furthermore, cleaned data were mapped for the analysis of annotation information, relative abundance tables, principal component analysis (PCA), heatmap generation, Kyoto Encyclopedia of Genes and Genomes (KEGG) homolog spectra, and pathway maps. The threshold for statistical significance was set at *p* < 0.05.

### 4.5. Validation by RT- qPCR

The mRNA levels of four up-regulated genes (AtWU_03490, AtWU_03807, AtWU_00075, and AtWU_10270) of *A. tubingensis* were analyzed using reverse transcription-quantitative PCR (RT-qPCR). The total RNAs were extracted from *A. tubingensis* using TRIzol^®^ reagent (Thermo Fisher Scientiffc, Inc., USA) according to the manufacturer’s protocol. The RNA integrity was assessed using the RNA Nano 6000 Assay Kit of the Bioanalyzer 2100 system (Agilent Technologies, Inc., Santa Clara, CA, USA). Subsequently, RNA was reverse transcribed into cDNA using a Prime-Script™ cDNA synthesis kit (Takara Bio, Inc., Beijing, China). RT-qPCR was performed on a CFX96 system (Bio-Rad Laboratories, Inc., Hercules, CA, USA) using Power SYBR Green RT-qPCR Master Mix (Roche Diagnostic, Shanghai, China) following the manufacturer’s protocol. The standard curves were constructed to determine the amplification efficiencies of the target and endogenous reference genes. The amplification effieciencies were comparable, and the ΔCT value for each sample was determined by calculating the difference between the CT value of the target gene and the CT value of the endogenous reference gene. Next, the ΔΔCT value for each sample was determined by subtracting the ΔCT value of the calibrator from the ΔCT value of the sample. The normalized target gene expression level in the samples was 2^−ΔΔCT^. Primers used were listed in Table 3. Gene relative expression was calculated using the comparative 2^−ΔΔCT^ method [50].

### 4.6. Dual-Luciferase Reporter Gene Assay

The pmir-GLO luciferase reporter plasmids and the pRK5-cMYC eukaryotic expression plasmids used in this study were obtained from South China Agricultural University. The transcription factor FacB was predicted to bind to the promoter region of the most upregulated gene of *A. tubingensis* (AtWU_03807). The pRK5-cMYC-FacB plasmids and pmir-GLO-03807 plasmids were constructed using the ClonExpress II One Step Cloning Kit (Vazyme, Nanjing, China), and then transfected into HEK293T cells to investigate the impact of transcription factor FacB on the promoter activity of AtWU_03807. Two days after transfection, cells were extracted and lysed. A luciferase gene assay was performed using a luciferase assay system from Promega (Madison, WI, USA) based on a dual-luciferase gene analysis method. Renilla luciferase was used for comparison. The extent of target reporter gene activation was calculated as the ratio of firefly luciferase to Renilla luciferase units.

### 4.7. Data Availability

Raw reads of *A. tubingensis* in this study have been deposited in the NCBI database URL (accessed on 26 June 2023) under the accession number PRJNA987324.

### 4.8. Statistical Analysis

All experiments were conducted in triplicate, and the statistical analyses were performed in GraphPad Prism 10 software. The data were presented as mean ± standard deviation. Differential comparisons were determined using Student’s *t*-test and one-way ANOVA analysis, with statistical significance defined as *p* < 0.05.

## 5. Conclusions

In conclusion, this study demonstrates that the transcriptional response to tannin biodegredation by *A. tubingensis* was highly influenced by different growth and cultivation conditions (solid/liquid). Liquid cultures induced wider transcriptomic variability compared to solid cultures during tannin biodegredation by *A. tubingensis*. These findings provide a better understanding of transcriptomic response to tannin biodegredation by *A. tubingensis* in different cultures.

## Figures and Tables

**Table 1:** Top 10 GO terms enriched in *A. tubingensis* grown in liquid culture.

	ID	Description *	Set Size	Category
GSEA	GO:0043933	Protein-containing complex subunit organization	488	Process
	GO:1903506	Regulation of nucleic acid-templated transcription	475	Process
	GO:2001141	Regulation of RNA biosynthetic process	475	Process
	GO:0006355	Regulation of transcription, DNA-templated	470	Process
	GO:0006396	RNA processing	466	Process
	GO:0042886	Amide transport	446	Process
	GO:0009892	Negative regulation of metabolic process	435	Process
	GO:0015833	Peptide transport	434	Process
	GO:0045184	Establishment of protein localization	432	Process
	GO:0005694	Chromosome	424	Component
ORA	GO:0006396	RNA processing	101	Process
	GO:0005783	Endoplasmic reticulum	100	Component
	GO:0006259	DNA metabolic process	97	Process
	GO:0005886	Plasma membrane	93	Component
	GO:0019438	Aromatic compound biosynthetic process	90	Process
	GO:0006355	Regulation of transcription, DNA-templated	87	Process
	GO:1903506	Regulation of nucleic acid-templated transcription	87	Process
	GO:2001141	Regulation of RNA biosynthetic process	87	Process
	GO:0051276	Chromosome organization	83	Process
	GO:0005694	Chromosome	82	Component

* A database of GO terms in *A. tubingensis* grown in liquid and solid cultures was used to recover the ID and description of the protein.

**Table 2:** Top 10 KEGG enrichment in *A. tubingensis* grown in liquid culture.

	ID	Description *	Set Size
GSEA	ko01110	Biosynthesis of secondary metabolites	326
	ko01120	Microbial metabolism in diverse environments	169
	ko05014	Amyotrophic lateral sclerosis	145
	ko05022	Pathways of neurodegeneration—multiple diseases	139
	ko05016	Huntington disease	127
	ko05010	Alzheimer disease	120
	ko01240	Biosynthesis of cofactors	119
	ko05012	Parkinson disease	110
	ko05020	Prion disease	105
	ko03010	Ribosome	104
ORA	ko01240	Biosynthesis of cofactors	37
	ko01230	Biosynthesis of amino acids	25
	ko04111	Cell cycle—yeast	21
	ko01200	Carbon metabolism	21
	ko03040	Spliceosome	18
	ko04113	Meiosis—yeast	17
	ko04141	Protein processing in endoplasmic reticulum	17
	ko04146	Peroxisome	16
	ko00230	Purine metabolism	16
	ko00500	Starch and sucrose metabolism	15

* A database of KEGG enrichment in *A. tubingensis* grown in liquid and solid cultures was used to recover the ID and description of pathways.

**Table 3:** Primers used for gene cloning and expression analysis.

Primer Name	NCBI Gene ID	Description	Product Size (bp)	Primer Sequence (5′-3′)
AtWU_03490	56002975	ABC multidrug transporter	128	F: GCGTTCACATTCGGCTGGAAG
				R: GAGTAGACCTTGGCGTTCATTGC
AtWU_03807	56003291	Ribonuclease III	101	F: GACACTTCAAGGCAACGAGCAATC
				R: ACTCTTTGACGGCATCCTGTTTTCC
AtWU_00075	55999564	Arabinogalactan en-do-1,4-beta-galactosidase A	129	F: GGCAAGACCAGCAACTATGACAAC
				R: CTCGTCCCAGTCCCATCCATTG
AtWU_10270	56009747	PeptidyI-tRNA hydrolase	110	F: GGATTTGTCGGTCTTGAGGATTGG
				R: CCTTCAACTCCTGTTCACTCATCTC
β-tubulin	37238	Beta-Tubulin	148	F: AGCAGATGTTCGACCCCAA
				R: TAGGTCTGGTTCTTGCTCTGGATG

F: forward primer, R: reverse prime.

## Data Availability

The original contributions presented in the study are included in the article; further inquiries can be directed to the corresponding authors.
